# Dilated cardiomyopathy due to hypocalcaemia: a case report

**DOI:** 10.1186/s13256-024-04505-3

**Published:** 2024-04-11

**Authors:** Nilushka Rupasinghe, Priyanga Ranasinghe, Leonard Wanninayake

**Affiliations:** 1 Ministry of Health, Colombo, Sri Lanka; 2 Department of Pharmacology, Faculty of Medicine, University of Colombo, Colombo, Sri Lanka

**Keywords:** Hypocalcaemic cardiomyopathy, Primary hypothyroidism, Dilated cardiomyopathy

## Abstract

**Background:**

Hypocalcaemia is a rare, but reversible, cause of dilated cardiomyopathy causing heart failure. Several case reports have been reported on reversible cardiomyopathy secondary to hypocalcaemia.

**Case presentation:**

We report a case of 54-year-old female Sri Lankan patient who presented with shortness of breath and was diagnosed with heart failure with reduced ejection fraction due to dilated cardiomyopathy. The etiology for dilated cardiomyopathy was identified as hypocalcemic cardiomyopathy, secondary to primary hypoparathyroidism, which was successfully treated with calcium and vitamin D replacement therapy.

**Conclusion:**

This adds to literature of this rare cause of reversible cardiomyopathy secondary to hypocalcemia reported from the South Asian region of the world. This case highlights the impact of proper treatment improving the heart failure in patients with hypocalcemic cardiomyopathy.

## Introduction

Dilated cardiomyopathy (DCM) is a non-ischaemic heart muscle disease with structural and functional myocardial abnormalities. The clinical picture of DCM is defined by left or biventricular dilatation and systolic dysfunction in the absence of coronary artery disease, hypertension, valvular disease, or congenital heart disease [1]. DCM has a prevalence of 1:2500 [2]. Several etiologies for DCM have been reported. About 30% of DCM is primary or familial with a genetic basis [2]. Several secondary causes for dilated cardiomyopathy have been identified, the most common being infective myocarditis, toxins including alcohol, chemotherapeutic agents, metals and other compounds, autoimmune causes, neuromuscular disorders, and endocrine disorders [1, 2]. Hypocalcemia is identified as a reversible cause of dilated cardiomyopathy [3–5].

Intracellular calcium concentration changes are essential for cardiac myocyte activity [4]. The increase of intracellular calcium concentration transiting through calcium channels is followed by calcium release from sarcoplasmic reticulum, its binding to the troponin–tropomyosin complex, and stimulation of the mutual binding of actin and myosin [4, 6]. Therefore, intracellular hypocalcemia interferes with cardiac myocyte activity. However, there are other mechanisms responsible for the cardiomyopathy in hypocalcemia of which some are yet to be fully understood [4].

The major causes of hypocalcemia that causes hypocalcemia related cardiomyopathy are hypoparathyroidism (86%), vitamin D deficiency (5%), pseudohypoparathyroidism (2%), and celiac disease (2%) [4]. Of the causes of hypoparathyroidism, the most commonly reported cause is primary hypoparathyroidism, followed by hypoparathyroidism secondary to surgery [4]. The most important clinically relevant feature of cardiomyopathy secondary to hypocalcemia is it can be reversed with proper treatment with calcium and vitamin D replacement [3–5, 7, 8]. We report a case of reversible dilated cardiomyopathy due to hypocalcemia secondary to primary hypoparathyroidism in a female Sri Lankan patient.

## Case report

A 54-year-old Sri Lankan patient with long standing type 2 diabetes mellitus, bronchial asthma, and hypothyroidism on treatment with metformin, thyroxin, and inhaled corticosteroids was admitted with sudden worsening of shortness of breath over 3 days. She denied any history of worsening angina, fever, cough, or wheezing.

She was dyspneic with orthopnea. She had gross bilateral ankle edema up to the mid calves, with a pulse rate of 100 per minute and a blood pressure of 100/70 mmHg. The cardiac apex was on the 6th intercoastal space lateral to the mid clavicular line. She had bilateral lung crepitations extending up to the upper zone without any evidence of bronchoconstriction or consolidation.

Her hemoglobin level was 13.6 g/dL, serum creatinine was 1.2 mg/gL, and serum albumin was 4.2 g/dL, all of which were in the normal range. Thyroid stimulating hormone (TSH) level was 3.5 mIU/L and was normal. Electrocardiogram (ECG) had only sinus tachycardia and no ischemic changes. Troponin I was negative on two tests. Brain natriuretic peptide (BNP) was 1200 pg/mL and was significantly elevated.

**Fig. 1:**
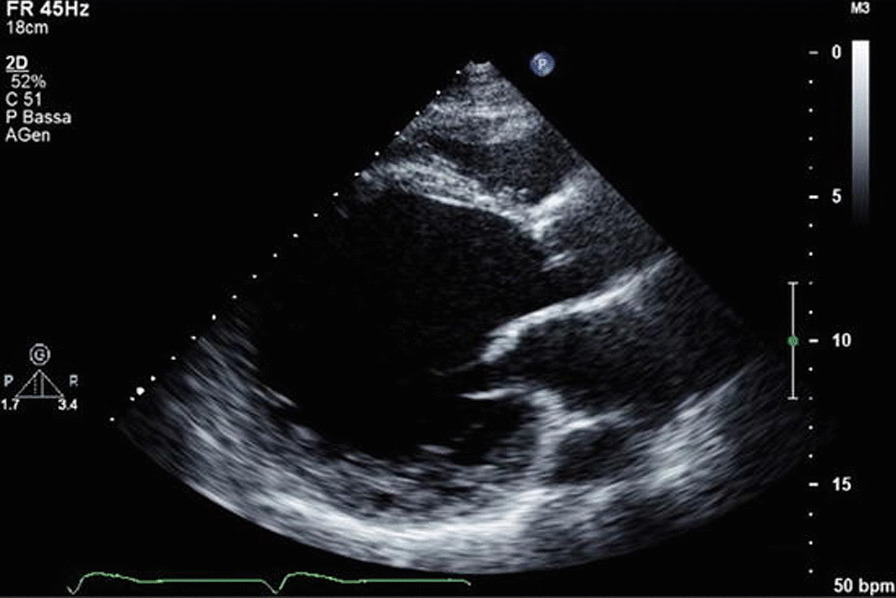
Two-dimensional echocardiogram showing evidence of dilated cardiomyopathy

Her two-dimensional echocardiogram revealed severely impaired left ventricular systolic function with an ejection fraction of 30%. All four chambers of the heart were dilated with severely impaired right ventricular function as well. There was a functional grade II mitral regurgitation and tricuspid regurgitation (Fig. 1). A clinical diagnosis of dilated cardiomyopathy with heart failure with reduced ejection fraction (HFrEF) was made.

On the acute presentation to the emergency treatment unit, the patient was managed with oxygen support with a continuous positive airway pressure (CPAP) machine and with intravenous frusemide bolus followed by an infusion. After the acute management, the patient was commenced on aspirin, atorvastatin, enalapril, and spironolactone. The patient clinically improved over a course of 3 days of inward stay.

Evaluating the cause of dilated cardiomyopathy with HFrEF, the patient did not give a family history suggestive of a similar cardiac illness. She had not consumed alcohol or other illicit drugs. There was no history of angina or other acute coronary events in the past. The coronary angiogram done a few days after stabilization revealed only minor coronary artery disease that cannot explain the current presentation due to obstructive coronaries. The patient was clinically euthyroid with normal thyroid-stimulating hormone (TSH). There was no history suggestive of myocarditis or other connective tissue disorders. However, the patient was detected to have a very low serum ionized calcium level of 2.0 mg/dL.

**Fig. 2:**
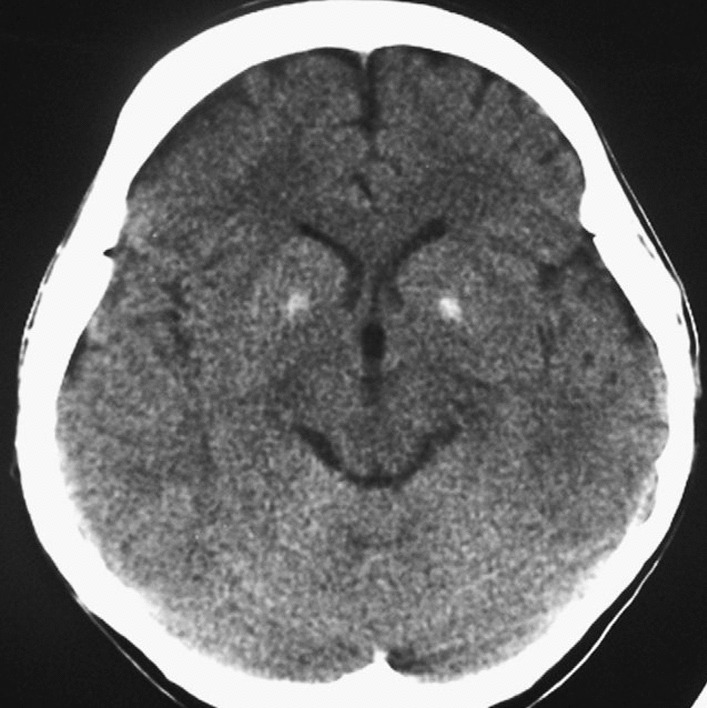
Showing evidence of basal ganglia calcifications in noncontrast computed tomography of the brain

Further evaluation of hypocalcemia revealed a very low level of intact parathyroid hormone (PTH) of 2 pg/mL. Serum 17 OH cholecalciferol level was low with 10 ng/mL. The non-contract computed tomography (CT) of the brain also showed basal ganglia calcifications, which supports the diagnosis of primary hypoparathyroidism (Fig. 2).

The patient was treated with high dose calcium and vitamin D therapy liasing with the endocrinology team. The calcium and vitamin D levels were assessed weekly during the first few weeks of treatment until achieving the normal range. Subsequently, the calcium level was monitored biweekly and monthly thereafter. Along with the treatment of hypocalcemia, she was also treated with pillars of HFrEF therapy, angiotensin converting enzyme inhibitor (enalapril), beta blocker (bisoprolol), and aldosterone antagonist (spironolactone). However, the patient was unable to afford sodium glucose cotransporter 2 (SGLT2) inhibitor therapy, as the medicine was not freely available in the state sector. Initially the patients symptoms, and the two-dimensional echo improvement was assessed in 2 week intervals, and then it was assessed monthly. After continuous follow-up with the therapy for hypocalcemia and the HFrEF, the patient clinically improved. In 3 months, the cardiac functions reverted back to a normal systolic function, with an ejection fraction of 55%.The patient is still on calcium supplements and her calcium level is monitored once a month with the assessment of her cardiac functions annually or sooner if any suggestive symptoms or signs develop.

## Discussion

Calcium is an integral ion in the cardiac myocyte contraction [4]. Intracellular calcium concentration changes are essential for cardiac myocyte activity [4]. During cardiac action potential, L-type Ca^2+^ channels are activated, and a majority of Ca^2+^ enters the cell through the Ca^2+^ current. Other exchanges, such as Na^+^–Ca^2+^ exchange also contributes for the intracellular Ca^2+^ influx. Influx of the intracellular Ca^2+^ triggers Ca^2+^ release from the sarcoplasmic reticulum. The net effect of increased intracellular Ca^2+^ is the binding of intracellular Ca^2+^ to multiple cytosolic Ca^2+^ buffers, of which the thin filament protein troponin C acts as a key cytosolic buffer. Binding of Ca^2+^ to troponin C activates the myofilaments to contraction. Similarly, during diastole, the intracellular Ca^2+^ concentration declines, enabling the Ca^2+^ to dissociate from troponin C, thereby stopping the contraction and enabling relaxation of the cardiac myocyte. There are four transporters that contribute in reduction of the concentration during diastole, namely: sarcoplasmic reticulum Ca^2+^–ATPase, sarcolemmal Na^+^–Ca^2+^ exchange, sarcolemmal Ca^2+^–ATPase, and mitochondrial Ca^2+^ uniporter [9]. It is evident from this physiology that calcium plays a vital role in both systolic contraction as well as diastolic relaxation of the cardiac myocyte. Thereby, hypocalcemia can result in severe dysfunction of cardiac myocyte, resulting in heart failure. However, there are other mechanisms responsible for the cardiomyopathy in hypocalcemia, of which some are yet to be fully understood [4].

The deficiency of calcium affects cardiac myocyte contraction, resulting in a rare form of reversible dilated cardiomyopathy causing heart failure with reduced ejection fraction, which has been reported in few cases around the world [3–5].

This case adds to the limited literature on reported case of reversible dilated cardiomyopathy secondary to hypocalcemia. Such cases reported in this region of the world are rare.

Previous literature reviews have identified the possible causes of hypocalcemia leading to cardiomyopathy, of which primary hypoparathyroidism being the commonest accounting for 86% of the reported cases [4]. This case also reported to have very low parathyroid hormone levels, with evidence of basal ganglia calcifications, which favors a diagnosis of primary hypoparathyroidism [10].

Given the history of hypothyroidism in this patient in the past and the newly diagnosed primary hypothyroidism, the possibility is raised of an autoimmune polyglandular syndrome in this patient. However, the absence of other endocrine manifestations and the manifestation of the disease in middle age, makes this rare diagnosis less likely in this case [11].

As reported in previous literature, the cardiomyopathy and the heart failure of this patient was reverted with the treatment of calcium and vitamin D supplementation along with the treatment of HFrEF. Therefore, the suspicion and the clinical vigilance on hypocalcemiais important in heart failure management as it is a reversible cause of heart failure.

## Conclusion

This is a rare case of reversible dilated cardiomyopathy with HFrEF in a middle-aged Sri Lankan patient secondary to primary hypoparathyroidism. The patient was successfully treated with calcium and vitamin D replacement, resulting in improvement of the heart failure and cardiomyopathy. This highlights the importance of being vigilant on this rare cause of heart failure as the proper diagnosis and treatment leads to reversal of the cardiac functions.

## Data Availability

Supporting data of the case is available.
